# Surfactant Protein (SP) induces preterm birth by promoting oxidative stress via upregulating Storkhead-Box Protein 1

**DOI:** 10.1016/j.clinsp.2022.100079

**Published:** 2022-09-07

**Authors:** Xiafang Li, Chunnian Zhang

**Affiliations:** a Department of Obstetrics, Ganzhou People's Hospital, Ganzhou, Jiangxi, China; b Department of Obstetrics and Gynecology, Ganzhou People's Hospital, Ganzhou, Jiangxi, China

**Keywords:** SP-A, STOX1, Overexpression, Preterm Birth

## Abstract

Preterm birth is the leading cause of infant mortality. The mechanisms that instigate preterm birth remain elusive and this makes it difficult to predict or prevent preterm birth. In this study, the authors found that SP-A induced pathological damage to the placenta and promoted preterm birth. Through mechanism, SP-A promoted the expression of STOX1 which further promoted the oxidative stress in the placenta by inhibiting the activities of a series of antioxidant enzymes including SOD, CAT and GSH-Px. SP-A also induced dysregulation of arginine metabolism by inhibiting NOS2 and ARG2. Overexpression of STOX1 aggravated SP-A induced oxidative stress, pathological damage, and preterm birth, whereas knockdown of STOX1 alleviated SP-A induced oxidative stress, pathological damage and preterm birth. The present study uncovers that SP-A induces preterm birth by promoting oxidative stress via upregulating STOX1, which provides new targets for the prediction and prevention of preterm birth.

## HIGHLIGHTS

• SP-A induced pathological damage to placenta by promoting STOX1.
• SP-A inhibited the activities of antioxidant enzymes through STOX1.
• SP-A promoted the ROS production in placenta by promoting STOX1.
• SP-A inhibited protein levels of NOS3 and ARG2 in placenta.

## Background

Preterm birth is defined as delivery before 37 weeks.^1^ It has been reported that the global preterm birth rates are up to ten percent.^2^ Preterm birth is a leading cause of neonatal death and those surviving infants have increased risks of inflammatory disorders, neurodevelopmental disorders, metabolic disorders, and early-life infections.^3^, ^4^, ^5^ The majority of preterm births are spontaneous and have no identifiable cause which limited the prediction or prevention of preterm birth. A detailed understanding of the molecular mechanisms underlying preterm birth is needed.

Maternal stress has been shown to be a risk factor for preterm birth.^6^ Various reports reported that there is a positive association between maternal stress and Corticotropin-Releasing Hormone (CRH) level and CRH appears to mediate the relationship between maternal stress and preterm birth.^7^, ^8^, ^9^ Placenta-derived CRH could promote the expression of cortisol and increased cortisol could promote fetal lung secret Surfactant Protein A (SP-A). SP-A could stimulate uterine contraction by promoting the production of prostaglandins and it can also initiate an inflammatory response to promote preterm birth.^10^, ^11^, ^12^

Oxidative stress, defined as dysregulation between antioxidants and oxidants, has been reported to contribute to the pathology of preterm birth.^13^ Excessive levels of ROS/RNS can cause cell necrosis, cell apoptosis, or cell senescence from protein alterations, lipid peroxidation, and DNA oxidation.^14^ Several studies have reported that preterm birth is associated with lower total antioxidant status and higher total oxidant status in the maternal blood and vaginal washing fluid.^15^, ^16^, ^17^ Oxidative stress is reported to induce damage to fetal membranes and placental cells which further generate uterotonic biomolecular signals that trigger the labor process.^13^

Storkhead Box 1 (STOX1) is a transcription factor that has been shown to be related to recurrent spontaneous abortion and pre-eclampsia.^18^^,^^19^ Overexpression of STOX1 could lead to transcriptome alterations involved in several cellular pathways and mitochondrial function is highly represented.^20^ STOX1 overexpression results in improved free radical production by inhibiting the expression of a series of important antioxidant modulators, and aggravating pre-eclampsia.^18^ However, the role of STOX1 in preterm birth has not been investigated.

In this study, the authors established a preterm birth model through intra-amniotic injection of SP-A in pregnant mice. The authors found that STOX1 was increased in the placenta in the preterm birth group. Knockdown of STOX1 could rescue SP-A-induced pathological damage to the placenta. By Mechanism, SP-A inhibited the activities or protein levels of a series enzymes including SOD, CAT, GSH-Px, NOS3, and ARG2 through promoting STOX1 and therefore caused increased oxidative stress in the placenta and promoted preterm birth. The present study recovers the role of STOX1 in preterm birth and provides a new target for predicting and preventing preterm birth.

## Methods

### Animals

BALB/c mice, aged 7‒8 weeks, were purchased from Charles River. The mice were housed in specific pathogen-free conditions at room temperature (22±3°C) and humidity (35%±5%) with a light-dark cycle. All animal experiments were approved by Ganzhou People's Hospital Ethics Committee and all animal experiments were performed in compliance with the guide for the care and use of laboratory animals.

### Induction of preterm birth

7‒8-week-old BALB/c female mice were co-housed with BALB/c male mice overnight and the presence of a copulatory plug was recorded as day 0 of gestation. The pregnant mice were randomly divided into four groups including the normal control group (NC group), SP-A group, SP-A+ov-STOX1 group, and SP-A+si-STOX1 group. To induce stress response, the mice in the SP-A group, SP-A+ov-STOX1 group and SP-A+si-STOX1 group were subjected to inverted light-dark cycles from day 10 to day 15 of gestation. The mice in the SP-A group, SP-A+ov-STOX1 group and SP-A+si-STOX1 group were given 3 ug SP-A (LSBio) through intra-amniotic injection at day 15 of gestation. Meanwhile, mice in SP-A+ov-STOX1 group were i.v. injected with ov-STOX1 and mice in SP-A+si-STOX1 group were i.v. injected with si-STOX1. After preterm delivery, the placenta of mice was collected and analyzed. Meanwhile, the placenta of mice in NC group was also collected and analyzed.

### Western blot

The placenta tissues were dissected from embryos in pre-cooled PBS in a 6 cm tissue culture dish and homogenized with a tissue homogenizer and radioimmune precipitation assay lysis buffer was used to extract protein. The protein samples were then separated with 10% SDS-PAGE and transferred to PVDF membranes. 5% bovine serum albumin was used to block PVDF membranes. The PVDF membranes were then incubated with primary anti-actin antibody, anti-STOX1 antibody, anti-NOS3 antibody, or anti-ARG2 antibody overnight and subsequently incubated with corresponding secondary antibodies. The protein bands were imaged using an ECL reagent (Bio‑Rad Laboratories, Inc.).

### Immunohistochemistry

Immunohistochemistry was used to determine the expression and location of STOX1 in the placenta. The placenta tissues were dissected from embryos in pre-cooled PBS in a 6 cm tissue culture dish and fixed with 4% PFA, paraffin-embedded, and sectioned into 5-μm sections. The sections were deparaffined with xylene and rehydrated in serial ethanol baths. Citrate buffer was used for antigen retrieval, 3% of hydrogen peroxide was used to block endogenous peroxidase and 5& BSA was used to block nonspecific binding of antibodies. The samples were incubated with anti-STOX1 primary antibody and subsequently incubated with secondary antibody. The DAB agent was used for the visualization of the expression of STOX1.

### H&E staining

The placenta tissues were dissected from embryos in pre-cooled PBS in a 6 cm tissue culture dish and fixed with 4% PFA, embedded with paraffin, and then sectioned into 5-μm sections. The samples were deparaffined with xylene and rehydrated in serial ethanol baths for hematoxylin and eosin staining. The sections were analyzed with a Light microscope (Olympus).

### Isolation of placental cells

The placenta tissues were dissected from embryos, cut into minor pieces, and then digested in complete RPMI 1640 medium containing 0.1 mg/mL DNAse I and 200 U/mL Type VIII collagenase at 37°C for 20 minutes with shaking at 220 rpm. The samples were then filtered through 70 μm cell strainer and centrifuged at 1000 rpm for 5 minutes. Placental cells were resuspended with a complete RPMI 1640 medium.

### ROS detection

The contents of Reactive Oxygen Species (ROS) were determined using CM-H2DCFDA kit according to manufacturer's instructions. Briefly, the placental cells were loaded with 1 uM CM-H2DCFDA for 30 min at room temperature. The cells were observed immediately under a fluorescent microscope (Olympus).

### Biochemical analysis

Placentas were homogenized and centrifuged and protein concentrations in the supernatants were measured by the method of Bradford. The activities of SOD, CAT, and GSH-Px in the supernatant were determined using a superoxide dismutase detection kit (njjcbio), a catalase detection kit (njjcbio), and Glutathione peroxidase detection kit (njjcbio) respectively. SOD activity was measured by detecting the inhibition of nitroblue tetrazolium (NBT) reduction by O_2_ ‒ generated from the xanthine/xanthine oxidase system. CAT activity was determined according to the method of Aebi by measuring the decomposition of H_2_O_2_ (66 mM) at 240 nm for 1 minute. The GSH-Px activity was determined based on the formation of a yellow-colored complex with Ellman's Reagent and the absorbance of which is at 412 nm.

### Statistical analysis

Data were presented as Mean ± SD. The student's *t*-test was used to analyze the difference between the two groups. Differences were considered significant at (*) p < 0.05, (**) p < 0.01 and (***) p < 0.001. Statistical analysis was conducted using GraphPad Prism software.

## Results

### SP-A induced pathological damage to placenta through promoting STOX1

**Figure 1:**
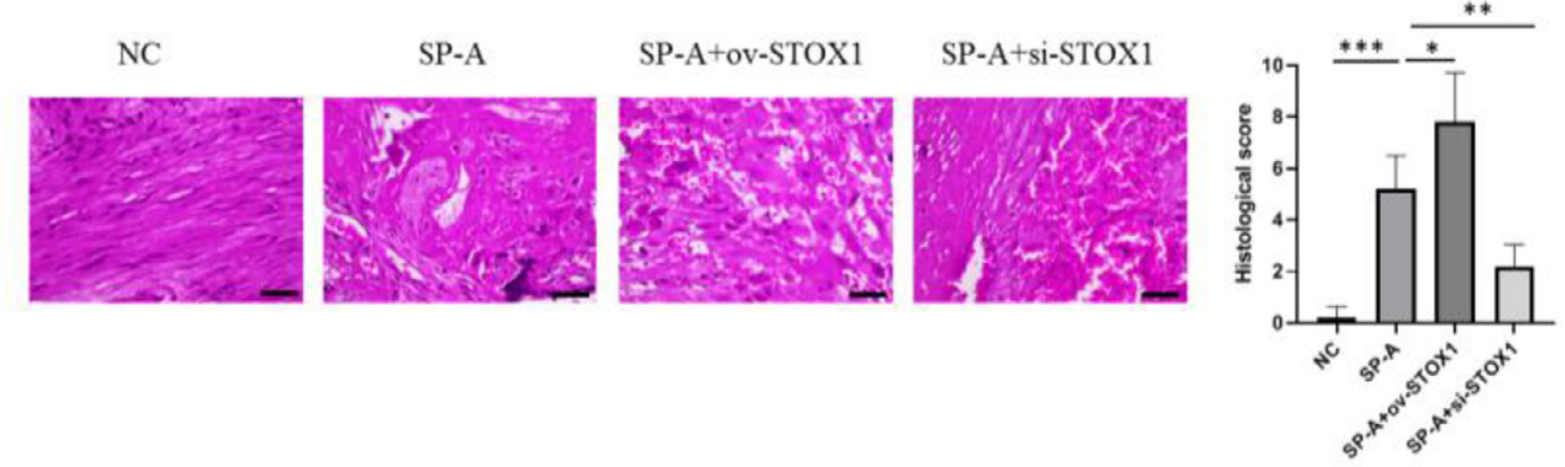
**SP-A induced pathological damage to placenta through promoting STOX1.** H&E staining images of placentas from NC group, SP-A group, SP-A+ov-STOX1 group and SP-A+si-STOX1 group (n = 6, Scale bar: 100 μm).

**Figure 2:**
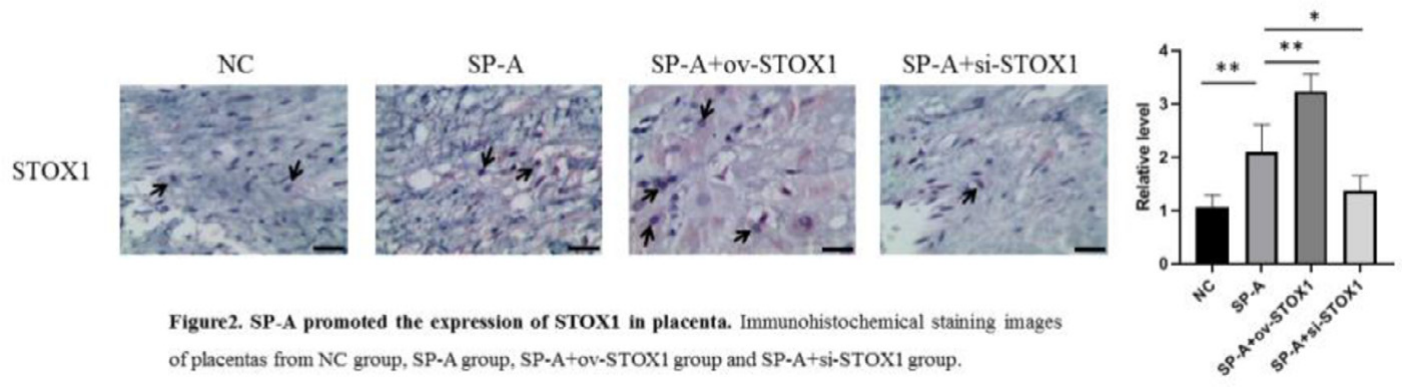
**SP-A promoted the expression of STOX1 in placenta.** Immunohistochemical staining images of placentas from NC group, SP-A group, SP-A+ov-STOX1 group and SP-A+si-STOX1 group. Arrows point to STOX1 in nucleus (n = 6, Scale bar: 100 μm).

It has been shown that SP-A could promote preterm birth.^12^ The authors established a mouse model of preterm birth through upregulating SP-A levels, housing mice under a reversed light/dark cycle to increase maternal stress to increase endogenous SP-A and intra-amniotic injection of SP-A to increase exogenous SP-A. Intraamniotic injection of SP-A caused preterm delivery of fetuses within 24h. After preterm birth, the placenta was isolated and analyzed through H&E staining. The authors found that SP-A induced pathological damage to the placenta indicated by fibrinoid necrosis of placental villi, liquefaction of interstitial effusion, nuclear embrittlement of trophoblasts, and congestion of spiral artery (Fig. 1). To investigate the role of STOX1 in SP-A induced preterm birth, ov-STOX1 was given to overexpress STOX1 while si-STOX1 was given to knockdown of STOX1. And the authors found that STOX1 overexpression aggravated the SP-A induced pathological damage to the placenta while knockdown of STOX1 alleviate the SP-A induced pathological damage to the placenta (Fig. 1). These results indicated that SP-A induced pathological damage to the placenta by promoting STOX1. To further validate the role of STOX1 in SP-A induced preterm birth, immunohistochemistry was performed to determine the expression of STOX1 in the placenta. The authors found that ov-STOX1 overexpressed STOX1 while si-STOX1 inhibited STOX1 in the placenta effectively. More importantly, the authors found that SP-A promoted the expression of STOX1 in the placenta. We also found that SP-A induced nuclear embrittlement of trophoblast, the disappearance of interstitial tissue, and excessive fibrin deposition in the placenta and which is aggravated through ov-STOX1 and alleviated through si-STOX1 (Fig. 2). These results further showed that SP-A promoted preterm birth through promoting STOX1.

### SP-A inhibited the activities of antioxidant enzymes through STOX1

**Figure 3:**
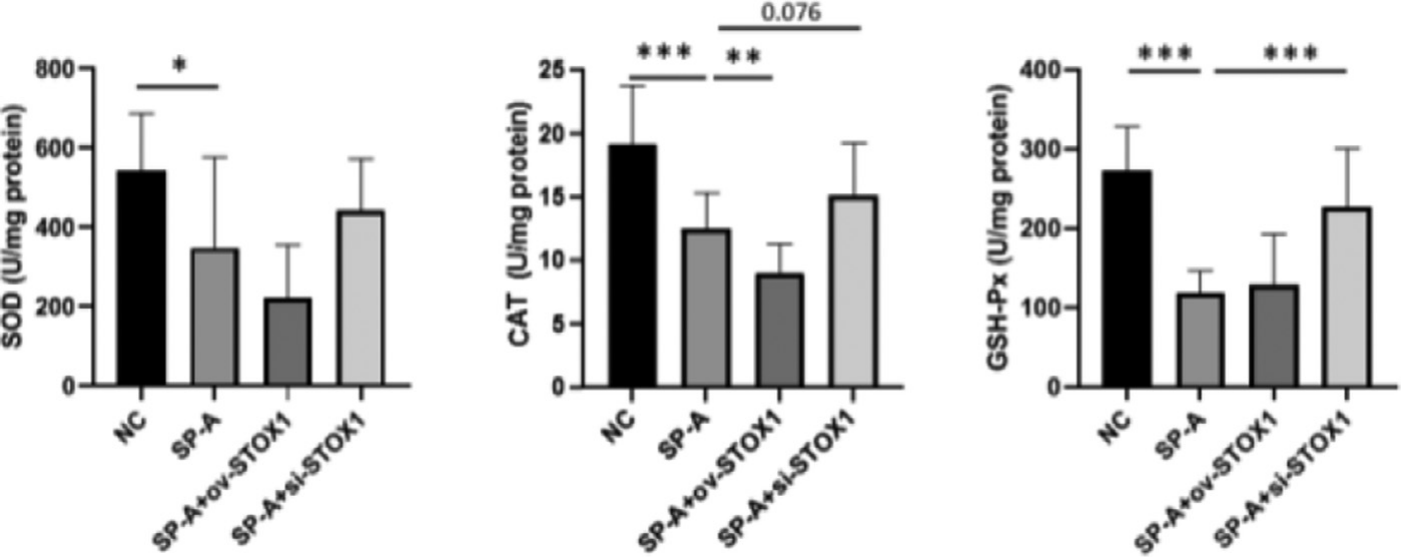
**SP-A inhibited the activities of antioxidant enzymes through promoting STOX1 in placenta.** The activities of SOD, CAT, GSH-Px and NOS in placentas from NC group, SP-A group, SP-A+ov-STOX1 group and SP-A+si-STOX1 group were shown (n = 6).

STOX1 overexpression has been reported to promote free radical production through inhibiting the expression of a series of important antioxidant modulators.^18^ And oxidative stress could result in cell apoptosis, cell necrosis and cell senescence and is related to multiple diseases.^13^^,^^21^^,^^22^ So we speculate that STOX1 may be involved in SP-A induced preterm birth through regulating oxidative stress. Superoxide Dismutase (SOD), Glutathione Peroxidase (GSH-Px), and Catalase (CAT) are well-known antioxidant enzymes that play important roles in the antioxidant system.^23^ So the authors investigated the activities of SOD, CAT, and GSH-Px in the placenta. The authors found that SP-A inhibited the activities of SOD, CAT, and GSH-Px. Moreover, STOX1 overexpression further inhibited the activities of these antioxidant enzymes while knockdown of STOX1 rescued the activities of SOD, CAT, and GSH-Px significantly (Fig. 3). These results indicated that SP-A inhibited the activities of SOD, CAT and GSH-Px through STOX1.

### SP-A promoted the ROS production in placenta through promoting STOX1

**Figure 4:**
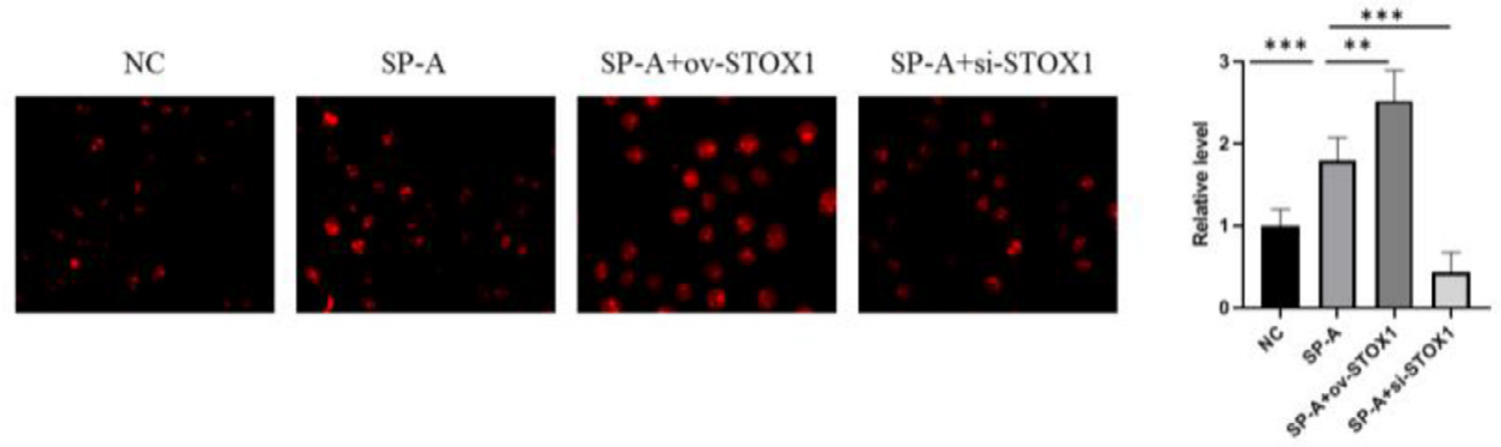
**SP-A promoted ROS production through promoting STOX1 in placenta.** Images of intracellular ROS production measured by CM-H2DCFDA in placenta from NC group, SP-A group, SP-A+ov-STOX1 group and SP-A+si-STOX1 group (n = 6).

The authors further investigated the ROS levels in placentas. The authors found that SP-A promoted ROS production in the placenta. STOX1 overexpression further promoted the ROS level, and the ROS level was decreased when STOX1 was inhibited (Fig. 4). These results, combined with Figure 3, suggested that SP-A increased oxidative stress in the placenta by inhibiting the activities of antioxidant enzymes via STOX1.

### SP-A inhibited protein levels of NOS3 and ARG2 in placenta

**Figure 5:**
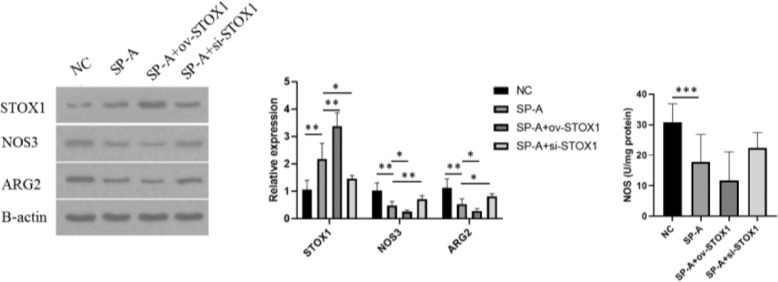
**SP-A inhibited NOS3 and ARG2 through promoting STOX1 in placenta.** Western blot was performed to determine the protein levels of STOX1, NOS3 and ARG2 in placentas from NC group, SP-A group, SP-A+ov-STOX1 group and SP-A+si-STOX1 group (n = 6)

The authors further investigated the protein levels of ARG2 and NOS3, two enzymes involved in arginine metabolism.^24^ The authors found that SP-A inhibited the protein levels of NOS3 and ARG2. STOX1 overexpression further inhibited the protein levels of NOS3 and ARG2 while knockdown of STOX1 rescued the protein levels of NOS3 and ARG2. The authors also detected the activity of NOS3. Consistently, the activity of NOS3 was inhibited by SP-A. STOX1 overexpression further inhibited the activity of NOS3 activity while knockdown of STOX1 rescued the activity of NOS3 (Fig. 5). These results indicated that SP-A resulted in dysregulation of arginine metabolism through promoting STOX1.

## Discussion

Preterm birth is a life-threatening disease and the surviving infants are at increased risk for multiple diseases.^25^^,^^26^ Despite some factors that have been shown to be related to preterm birth, the majority of preterm births are spontaneous and have no identifiable cause.^27^^,^^28^ Detailed understanding of the molecular mechanisms underlying preterm birth is important for the prediction and prevention of preterm birth. In this study, the authors established a mouse model of preterm birth through intra-amniotic injection of SP-A. The authors found that SP-A induced pathological damage to the placenta. By mechanism, SP-A promoted the expression of STOX1 and STOX1 further promoted the oxidative stress in the placenta through inhibiting the activities of antioxidant enzymes. The present findings reveals a new mechanism underlying preterm birth and provides new targets for the prediction and prevention of preterm birth.

The authors found that SP-A promoted the expression of STOX1 which further inhibited the activities of antioxidant enzymes, SOD, CAT, and GSH-Px. But how STOX1 regulates the activities of SOD, CAT and GSH-Px need to be further investigated. The authors speculate that as a transcription factor, STOX1 may promote the expression of some microRNAs, and these microRNAs targets and downregulate the expression of antioxidant enzymes. The decreased protein level of these antioxidant enzymes could result in downregulated activities in tissue homogenate. on the other hand, STOX1 may also bind with these antioxidant enzymes directly and inhibit their activities. Further study will help to recover the mechanisms underlying the effects of STOX1 on antioxidant enzymes.

## Authors' contributions

Each author has made an important scientific contribution to the study and has assisted with the drafting or revising of the manuscript.

## Ethics approval and consent to participate

The ethical approval was obtained from the Ethics Committee of Ganzhou People's Hospital.

## Consent to publish

All of the authors have Consented to publish this research.

## Availability of data and materials

The data are free to access to available upon request.

## Funding

None.

## Conflicts of interest

The authors declare no conflicts of interest.
